# Playing Atari with few neurons: Improving the efficacy of reinforcement learning by decoupling feature extraction and decision making

**DOI:** 10.1007/s10458-021-09497-8

**Published:** 2021-04-19

**Authors:** Giuseppe Cuccu, Julian Togelius, Philippe Cudré-Mauroux

**Affiliations:** 1 eXascale Infolab, Department of Computer Science, University of Fribourg, Fribourg, Switzerland; 2 Game Innovation Lab, Tandon School of Engineering, New York University, New York, NY USA

**Keywords:** Game playing, Neuroevolution, Evolutionary algorithms, Learning agent capabilities

## Abstract

We propose a new method for learning compact state representations and policies separately but simultaneously for policy approximation in vision-based applications such as Atari games. Approaches based on deep reinforcement learning typically map pixels directly to actions to enable end-to-end training. Internally, however, the deep neural network bears the responsibility of both extracting useful information and making decisions based on it, two objectives which can be addressed independently. Separating the image processing from the action selection allows for a better understanding of either task individually, as well as potentially finding smaller policy representations which is inherently interesting. Our approach learns state representations using a compact encoder based on two novel algorithms: (i) Increasing Dictionary Vector Quantization builds a dictionary of state representations which grows in size over time, allowing our method to address new observations as they appear in an open-ended online-learning context; and (ii) Direct Residuals Sparse Coding encodes observations in function of the dictionary, aiming for highest information inclusion by disregarding reconstruction error and maximizing code sparsity. As the dictionary size increases, however, the encoder produces increasingly larger inputs for the neural network; this issue is addressed with a new variant of the Exponential Natural Evolution Strategies algorithm which adapts the dimensionality of its probability distribution along the run. We test our system on a selection of Atari games using tiny neural networks of only 6 to 18 neurons (depending on each game’s controls). These are still capable of achieving results that are not much worse, and occasionally superior, to the state-of-the-art in direct policy search which uses two orders of magnitude more neurons.

## Introduction

In deep reinforcement learning, a large network learns to map complex, high dimensional input (often visual) to actions, for direct policy approximation. When a giant network with hundreds of thousands of parameters learns a relatively simple task (such as playing the classic action-puzzle game *Qbert*) it stands to reason that only a small part of what is learned is the actual policy. A common understanding is that the network internally learns to extract useful information (features) from the image observation in its first layers by mapping pixels to intermediate representations, allowing the last few layer(s) to map these representations to actions. The fact that such intermediate representations are learned by deep neural nets is borne out by numerous studies on supervised learning, though the issue is less well-studied in reinforcement learning. Learning the policy at the same time as the intermediate representations makes it almost impossible to study the policy in isolation.

Separating representation learning from policy learning allows in principle for higher component specialization, enabling smaller networks dedicated to policy learning to address problems typically tackled by much larger networks [1, 10, 27]. This size difference represents a net performance gain, as larger networks can be devoted to addressing problems of higher complexity. For example, current results on Atari games are achieved using networks of hundreds of neurons and hundreds of thousands of connections; making the same game playable (with not much lower performance) by a network *k* times smaller paves the way to training larger networks on *k* independent games, using currently available methods and resources.

Separating the policy network from the image parsing also allows to better understand how network complexity contributes to accurately representing the policy. While vision-based tasks are often addressed with very large networks, the learned policies by themselves should in principle not require such high-capacity models, as these policies in themselves often appear to not be very complex. Yet another reason to investigate how to learn smaller policy networks by addressing the image processing with a separate component is that smaller networks may offer better generalization. This phenomenon is well-known from supervised learning, where smaller-capacity models tend to overfit less, but has not been explored much in the context of reinforcement learning.

The key contribution of this paper is a new method for learning policy and features *simultaneously* but *separately* in a complex reinforcement learning setting. This is achieved by delegating feature extraction to two novel algorithms (namely Increasing Dictionary Vector Quantization (IDVQ) and Direct Residuals Sparse Coding (DRSC)), which leaves the neural network to specialize on policy approximation using neuroevolution. Without those two algorithms, performance on all games we played plateaued (e.g., we could not score higher than 640 on Qbert). Leveraging those two algorithms allow us to be much more competitive while considering surprisingly small networks as we will show in Sect. 5.

IDVQ maintains a dictionary of centroids in the observation space, which can then be used for encoding. The two main differences with standard VQ are that the centroids are (i) trained online by (ii) disregarding reconstruction error. Online training is achieved with the algorithm autonomously selecting images for its training from among the observations it receives to be encoded, obtained by the policies as they interact with the environment. The disregard for reconstruction error comes instead from shifting the focus of the algorithm to the arguably more crucial criterion (from the perspective of the application at hand) of ensuring that all of the information present in the observation is represented in the centroids. This is done by means of constructing new centroids as a residual image from the encoding while ignoring *reconstruction artifacts*. See Sect. 3.2 for further discussion.

The dictionary trained by IDVQ is then used by DRSC to produce a compact code for each observation. This code will be used in turn by the neural network (policy) as input to select the next action. The code is a binary string: a value of ‘1’ indicates that the corresponding centroid contains information also present in the image, and a limited number of centroids are used to represent the totality of the information.

As the training progresses and more sophisticated policies are learned, complex interactions with the environment result in increasingly novel observations; the dictionary reflects this by *growing in size*, including centroids that account for newly discovered features. A larger dictionary corresponds to a larger code, forcing the neural network to grow in input size. This is handled using a specialized version of Exponential Natural Evolution Strategy which adapts the dimensionality of the underlying multivariate Gaussian.

Experimental results show that this approach can effectively learn both components simultaneously, achieving performance similar to other evolutionary approaches (and not much lower than state-of-the-art approaches) on several ALE games while using a neural network of *only 6 to 18 neurons*, i.e. **two orders of magnitude smaller** than any known previous implementation.

This article is an extended version of our earlier paper, “Playing Atari with Six Neurons”, which appeared at AAMAS 2019 [11]. The present article features a larger set of experiments using additional games, more figures, a new discussion section, an example of limitations of the feature extraction, and additions to the introduction, background and related work sections.

## Related work

### Video games as AI benchmarks

Games are useful as AI benchmarks as they are designed to challenge human cognitive capacities. Board games such as Chess and Go have been used as AI benchmarks since the inception of artificial intelligence research, and have been increasingly used for testing and developing AI methods [48]. Various video game-based AI competitions and frameworks exist, based on games of different types and ages. Many are based on 2D arcade-style games, such as the Mario AI benchmark, based on a clone of the classic platformer Super Mario Bros [44]. The General Video Game AI (GVGAI) benchmark features more than a hundred 2D games, with the ability to easily change these games and create new ones [36]. Others are based on 3D games, for example VizDoom which is based on the classical FPS *Doom* [26], and *Obstacle Tower*, which is a state-of-the-art 3D platformer developed specifically to challenge AI agents in various ways [23]. There are also benchmarks based on strategy games such as StarCraft [32, 45]. All of these games pose different challenges to AI agents, and in general a model-free solution that works for one of them needs to at the very least to be re-trained to work for another (for model-based planning methods the situation is different, where MCTS-based agents can do well on many games; see recent progress on the GVGAI benchmark [35]). 2D games offer different perceptual and navigational challenges from 3D games; in some ways navigating a static 2D world can be harder than navigating a 3D first-person perspective, as it requires identifying the player avatar on screen and planning in static 2D space.

One very popular 2D game-based AI environment and benchmark is the Arcade Learning Environment (ALE), the introduction of which did much to catalyze the use of arcade games as AI benchmarks [3]. ALE is based on an emulation of the Atari 2600, the first widely available video game console with exchangeable games, released in 1977. This was a very limited piece of hardware: 128 bytes of RAM, up to 4 kilobytes of ROM per games, no video memory, and an 8-bit processor operating at less than 2 MHz. The limitations of the original game console mean that the games are visually and thematically simple. Most ALE games feature two-dimensional movement and rules mostly triggered by sprite intersection. In the most common setup, the raw pixel output of the ALE framework is used as inputs to a neural network, and the outputs are interpreted as commands for playing the game. No fast forward model is available, so planning algorithms are ineffective. Using this setup, Mnih et al. reached above human level results on a majority of 57 Atari games that come with the standard distribution of ALE [31]. Since then, a number of improvements have been suggested that have improved game-playing strength on most of these games [20, 25].

### Neuroevolution

Neuroevolution refers to the use of evolutionary algorithms to train neural networks [13, 21, 37, 49]. Typically, this means training the connection weights of a fixed-topology neural network, though some algorithms are also capable of evolving the topology at the same time as the weights [41].

When using neuroevolution for reinforcement learning, a key difference is that the network is only trained in between episodes, rather than at every frame or time step. In other words, learning happens between episodes rather than during episodes; this has been called *phylogenetic* rather than *ontogenetic* reinforcement learning [43]. While it could be argued that evolutionary reinforcement learning should learn more slowly than ontogenetic approaches such as Q-learning, as the network is updated more rarely and based on more aggregated information, the direct policy search performed by evolutionary algorithms allows in principle for a freer movement in policy space. Empirically, neuroevolution has been found to reach state-of-the-art performance on reinforcement learning problems which can be solved with small neural networks [15] and to reach close to state-of-the-art performance on games in the ALE benchmark played with visual input [5, 38]. In general, neuroevolution performs worse in high-dimensional search spaces such as induced by deep neural networks, but there have also been recent results where genetic algorithms have been shown to be competitive with gradient descent for training deep networks for reinforcement learning [42]. Neuroevolution has also been found to learn high-performing strategies for a number of other more modern games including racing games and first-person shooters, though using human-constructed features [37].

For training the weights of a neural network only, modern variants of evolution strategies can be used. The Covariance Matrix Adaptation Evolution Strategy (CMA-ES) [18] represents the population implicitly as a distribution of possible search points; it is very effective at training small-size networks in reinforcement learning settings [21]. Another high-performing development of evolution strategies is the Natural Evolution Strategies (NES) family of algorithms [47]. While both CMA and NES suffer from having a number of parameters required for evolution growing superlinearly with the size of the neural network, there are versions that overcome this problem [9, 39].

### Compressed representation in reinforcement learning

The high dimensionality of visual input is a problem not only for evolutionary methods, but generally for learning technique. The origin of the success of deep learning can be traced to how deep convolutional networks handle large dimensional inputs; up until a few years ago, reinforcement learning generally relied on low-dimensional features, either by using intrinsically low-dimensional sensors (such as infrared or laser range-finders) or by using hard-coded computer vision techniques to extract low-dimensional state representations from image data. Such hard mappings however do not lend themselves to generalization; in order to create a more general reinforcement learning method, the mapping must be automatically constructed or learned.

It has been suggested that problems have an “intrinsic” dimensionality, meaning how many parameters are needed in a model that learns to solve the problem [28]. However, this is in itself highly dependent on model architecture, as we shall see.

Several approaches have been proposed that use some kind of preprocessing to create a smaller input space for reinforcement learning. Some of them rely on neural networks, in particular on various forms of autoencoders [1, 17]. Togelius and Alvernaz present a method were an autoencoder to encode a small (approximately dimension 100) encoding of the visual input from the 3D game *DOOM*; the output from the encoder is fed into a smaller network trained through neuroevolution, and the autoencoder is continually retrained as the agent encounters visually novel situations [1]. Ha and Schmidhuber also used an autoencoder to learn a low-dimensional embedding of a pixel-based view, but then also learned a state transition model in the embedding space, and used neuroevolution to learn to play games in this space. An example of a neural compressor for Rl that does not rely on gradient descent is that of Koutnik et al [27], where a convolutional network was evolved to maximize output variance over a set of images. But neural networks such as autoencoders is not the only way to compress visual input. An alternative is to use external compressors, for example based on vector quantization [10], where a number of prototype vectors are found and each vector is used as a feature detector–the value of that feature being the similarity between the actual high-dimensional input and the vector, similar to a radial basis function network.

## Method

**Fig. 1:**
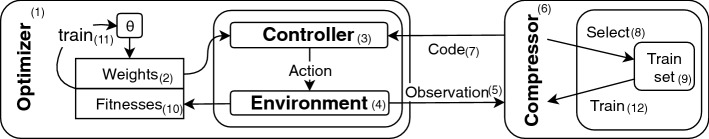
System diagram. At each generation the optimizer (1) generates sets of weights (2) for the neural network controller (3). Each network is evaluated episodically against the environment (4). At each step the environment sends an observation (5) to an external compressor (6), which produces a compact encoding (7). The network uses that encoding as input. Independently, the compressor selects observations (8) for its training set (9). At the end of the episode, the environment returns the fitness (cumulative reward; 10) to the optimizer for training (neuroevolution; 11). Compressor training (12) takes place in between generations

Our system is divided into four main components (see Fig. 1): i) the Environment is an Atari game, taking actions and providing observations; ii) the Compressor extracts a low-dimensional code from the observation, while being trained online with the rest of the system; iii) the Controller is our policy approximizer, i.e. the neural network; finally iv) the Optimizer is our policy learning algorithm, improving the performance of the network over time, in our case an Evolution Strategy. Each component is described in more detail below.

### Environment

We test our method on the Arcade Learning Environment (ALE), interfaced through the OpenAI Gym framework [4]. As discussed above, ALE is built on top of an emulator of the Atari 2600, with all the limitations of that console. In keeping with ALE conventions, the observation consists of a $[210 \times 180 \times 3]$ tensor, representing the RGB pixels of the screen input. The output of the network is interpreted (using one-hot encoding) as one of 18 discrete controls, representing the potential inputs from the joystick. The frame-skipping is fixed at 5 by following each action with 5 NOOP commands.

### Compressor

The role of the compressor is to provide a compact representation for each observation coming from the environment, enabling the neural network to entirely focus on decision making. This utilizes unsupervised learning on the very same observations that are obtained by the network interacting with the environment, in an *online learning* fashion.

We address such limitations through a new algorithm based on Vector Quantization (VQ), named Increasing Dictionary VQ, coupled with a new Sparse Coding (SC) method named Direct Residuals SC. Together they aim at supporting the study of the spaces of observations and features, while offering top performance for online learning. While this is not the first work to employ unsupervised learning as a pre-processor for neuroevolution [1, 10], the literature on the subject is fairly limited. The following sections will derive IDVQ+DRSC starting from the vanilla VQ, explaining the design choices which led to these algorithms

#### Vanilla vector quantization

The standard VQ algorithm [16] is a dictionary-based encoding technique with applications in dimensionality reduction and compression. Representative elements in the space (called singularly *centroids* and collectively called a *dictionary*) act as references for a surrounding volume, in a manner akin to k-means. The *code* of an element in the space is then a vector where each position corresponds to a centroid in the dictionary. Its values are traditionally set to zeros, except for the position corresponding to the closest representative centroid in the space. Variations use a dense code vector, capturing the contribution of multiple centroids for higher precision. In either case the original can be reconstructed as a vector product between the code and the dictionary. The difference between the original and its reconstruction is called *reconstruction error*, and quantifies the information lost in the compression/decompression process. The dictionary is trained by adapting the centroids to minimize reconstruction error over a training set.

Applications to *online* reinforcement learning however present a few limitations. Additional training data is not only unavailable until late stages, but is also only accessible if obtained by individuals through interaction with the environment. Take for example an Atari game with different enemies in each level: observing a second-level enemy depends on the ability to solve the first level of the game, requiring in turn the compressor to recognize the first-level enemies. A successful run should thereby alternate improving the dictionary with improving the candidate solutions: at any stage, the dictionary should provide an encoding supporting the development of sophisticated behavior.

In online learning though, two opposite needs are in play: on one hand, the centroids need to be trained in order to provide a useful and consistent code; on the other hand, late stage training on novel observations requires at least some centroids to be preserved untrained. Comparing to vanilla VQ, we cannot use random centroids for the code. As they are uniformly drawn from the space of *all possible images*, their spread is enormously sparse w.r.t. the small sub-volume of an Atari game’s image. The similarity of a random centroid to any such image will be about the same: using random centroids as the dictionary consequently produces an *almost constant* code for any image from a same game.^1^ Image differentiation is relegated to the least significant digits, making it suboptimal as a neural network input. Directly addressing this problem naturally calls for starting with a smaller dictionary size, and increasing it at later stages as new observations call for it.
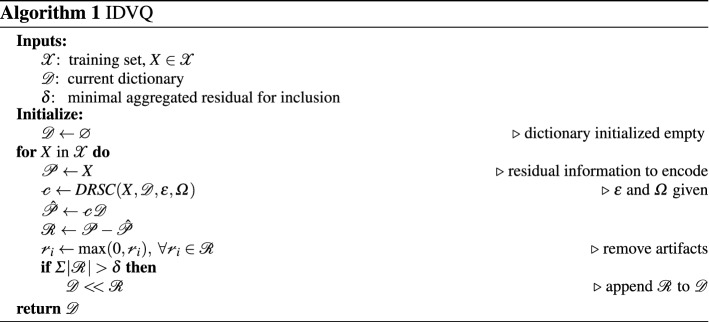


#### Increasing dictionary VQ

We introduce Increasing Dictionary VQ (IDVQ, Algorithm 1), a new compressor based on VQ which automatically increases the size of its dictionary over successive training iterations, specifically tailored for online learning. Rather than having a fixed-size dictionary, IDVQ starts with an *empty* dictionary, thus requiring *no initialization*, and adds new centroids as the learning progresses.

This is done by building new centroids from the positive part of the reconstruction error, which corresponds to the information from the original image (rescaled between 0 and 1) which is not reconstructed by the current encoding (see Algorithm 1). Growth in dictionary size is regulated by a threshold $\delta$, indicating the minimal aggregated residual considered to be a meaningful addition. The training set is built by uniformly sampling the observations obtained by all individuals in a generation.

Centroids added to the dictionary are not further refined. This is in line with the goal of image differentiation rather than minimizing reconstruction error: each centroid is purposely constructed to represents one particular feature, which was found in an actual observation and was not available in the dictionary before.

Growing the dictionary size however alters the code size, and thus the neural network input size. This requires careful updates in both the controller and the optimizer, as addressed in Sects. 3.3 and 3.4 respectively.
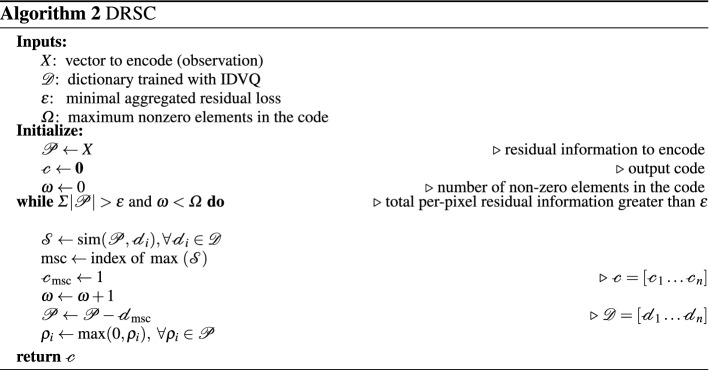

**Fig. 2:**
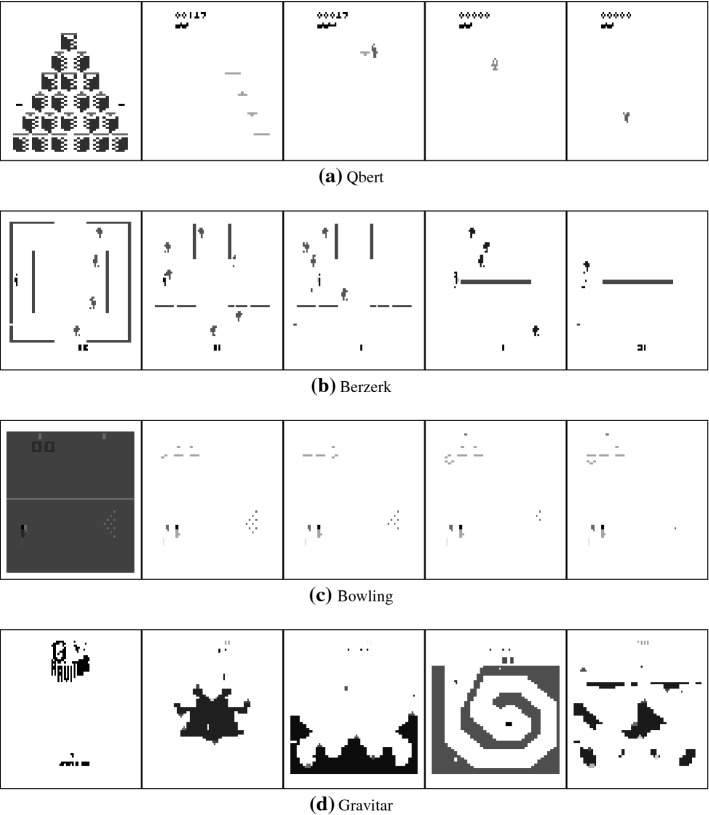
Trained centroids. Samples of centroids trained with IDVQ during runs on different games. Notice how the first centroid typically captures the initial state of the game, often identifiable as the background. By design, the following centroids then represent sprites that have changed w.r.t. that first image, thus identifying active elements of the game, such as avatars, enemy, and interactive props. Colors are inverted for printing convenience

#### Direct residuals sparse coding

The performance of algorithms based on dictionary approaches depends more on the choice of encoding than on the dictionary training—to the point where the best performing algorithms have but a marginal improvement in performance when using sophisticatedly trained centroids versus randomly selected samples [6]. This highlights the importance of selecting an effective encoding algorithm to best leverage the characteristics of a dictionary trained with IDVQ. In recent years, several studies have shown algorithms based on *Sparse Coding* to consistently perform best on compression and reconstruction tasks [29, 50]. These typically alternate training the centroids and minimizing the $\ell _1$ norm of the code (which approximates $\ell _0$ norm, i.e., the number of nonzero elements), ultimately yielding a code that is mostly composed of zeros. In our case though, the dictionary is already trained with IDVQ: we thereby focus on the construction of the sparse code instead.

The classic way to construct a sparse code is through an iterative approach [30, 34] where at each step (i) few centroids are selected, (ii) a corresponding code is built and (iii) the code quality is evaluated based on the reconstruction error, with the $\ell _1$ norm of the code as a regularization term. This process is repeated over different combinations of centroids to incrementally reduce the reconstruction error, at the cost of the algorithm’s performance. Moreover, the reconstruction is computed as a vector product between the code and the dictionary: while conceptually elegant, this dot product produces a linear combination (of the centroids with the code values) where most terms have null coefficients.

In our case though the focus is in differentiating states in order to support the decision maker, rather than perfecting the reconstruction of the original input. The encoding algorithm will be called on each and every observation coming from the environment, proportionally reducing the computational time available for decision making. This forces an overhaul of the encoder’s objective function from the ground up, prioritizing *distinction over precision*, i.e. observation differentiation over reconstruction error.

To this end we introduce Direct Residuals Sparse Coding (DRSC, Algorithm 2) as a novel sparse coding algorithm specifically tailored to produce highly differentiating encoding in the shortest amount of time. Its key characteristics are: (i) it utilizes centroids constructed as *residual images* from IDVQ, thus avoiding the centroid-train phase; (ii) it produces binary encodings, reducing the reconstruction process to an unweighted sum over the centroids corresponding to the code’s nonzero coefficients; and (iii) it produces the code in a single pass, terminating early after a small number of centroids are selected. The result is an algorithm with linear performance over dictionary size, which disassembles an observation into its consecutive most similar components as found in the dictionary.

#### Step-by-step breakdown

Increasing Dictionary VQ is used to train a dictionary, used by Direct Residuals SC to encode (compress, extract features from) an observation (image). To understand how these algorithms work together, let us hypothesize a working starting dictionary and see how DRSC produces an encoding.

The initialization includes two steps: the code, as an arrays of zeros with the same size as the dictionary, and the *residual information* still needing encoding, initially the whole original image. The algorithm then loops to select centroids to add to the encoding, based on how much of the residual information can they encode. To select the most similar centroid, the algorithm computes the differences between the residual information and each centroid in the dictionary, aggregating each of these differences by summing all values. The centroid with the smallest aggregated difference is thereby the most similar to the residual information, and is chosen to be included in the encoding. The corresponding bit in the binary code is flipped to ‘1’, and the residual information is updated by subtracting the new centroid.

The signs of the values in the updated residual information (old residual minus new centroid, the order matters) are now significant: (i) values equal to zero mean a perfect correspondence between the pixel information in the old residual and the corresponding value in the new centroid; (ii) positive values correspond to information that was present in the old residual but not covered by the new centroid; (iii) negative values correspond to information present in the new centroid, but absent (or of smaller magnitude) in the old residual. This is crucial towards the goal of fully representing the totality of the original information, and to this end the algorithm is free to disregard *reconstruction artifacts* as found in (iii).

Most encoding algorithms make no distinction between not-yet-encoded information and reconstruction artifacts: as they aim at minimizing reconstruction error, they focus on the error’s magnitude rather than its origin. DRSC instead focuses solely on representing all the information initially present in the image, and the artifacts found in the negative values are thereby disregarded by setting them to zero. The result is a residual image of information present in the original image but not yet captured by the reconstruction.

The algorithm then keeps looping and adding centroids until the (aggregated) residual information is lower than a threshold, corresponding to an arbitrary precision in capturing the information in the original image. To enforce sparsity in the case that the correct centroids are not available in the dictionary, a secondary stopping criterion for the encoding loop is when too many centroids are added to the code, based on another threshold. Images with high residual information after encoding are prime candidates for compressor training.

The dictionary is trained with IDVQ by adding new centroids to minimize leftover residual information in the encoding. The training begins by selecting an image from the training set and encoding it with DRSC, producing the binary code as described above. A dot product between the code and the dictionary (i.e. summing the centroids selected by the code, since it is binary) produces a reconstruction of the original image, similarly to other dictionary-based algorithms.

The difference between the training image and the reconstruction then produces a reconstruction error (-image), where the sign of the values once again correspond to their origin: positive values are leftover information from the image which is not encoded in the reconstruction, while negative values are reconstruction artifacts with no relation to the original image. This reconstruction error image is then aggregated (with a sum) to estimate the quantity of information missed by the encoding. If it is above a given threshold, a new centroid should be added to the dictionary to enable DRSC to make a more precise reconstruction. But in that case the residual itself makes for the perfect centroid, as it exactly captures the information missed by the current encoding, and is then added to the dictionary. In that sense, our algorithm does not directly differentiate between levels, but between observations. If the new level has the same visual representation, plus a constant (e.g. color shift, but same map), then a new centroid is added which captures the new constant shift, while the rest of the centroids remain relevant (as does the learned policy).

### Controller

The controller for all experiments is a single-layer fully-connected recurrent neural network (RNN). Each neuron receives the following inputs through weighted connections: the inputs to the network, the output of all neurons from the previous activation (initially zeros), and a constant bias (always set to 1). The number of inputs is equal at any given point in time to the size of the code coming from the *compressor*. As the compressor’s dictionary grows in size, so does the network’s input. In order to ensure continuity in training (i.e. the change needs to be transparent to the training algorithm), it is necessary to define an invariance across this change, where the network with expanded weights is equivalent to the previous one. This is done by setting the weights of all new connections to zero, making the new network mathematically equivalent to the previous one, as any input on the new connections cancels out. The same principle can be ported to any neural network application.

The number of neurons in the output layer is kept equal to the dimensionality of the action space for each game, as defined by the ALE simulator. This is as low as 6 in some games, and 18 at most. Actions are selected deterministically in correspondence to the maximum activation. No hidden layer nor extra neurons were used in any of the presented results. The increase in dimensionality in the input connections’ weights corresponds to a growth in the parameter vector of the optimizer, as described below in Sect. 3.4.2.

### Optimizer

The optimizer used in the experiments is a variation of Exponential Natural Evolution Strategy(XNES; [14]) tailored for evolving networks with dynamic varying size.

The next section briefly introduces the base algorithm and its family, followed by details on our modifications.

#### Exponential NES

Natural Evolution Strategies (NES; [46, 47]) is a family of evolutionary strategy algorithms that maintain a *distribution* over the parameters space rather than an explicit population of individuals. It is distinguishable over similarly distribution-based ES (e.g. Covariance Matrix Adaptation Evolution Strategy; CMA-ES [18]) for its update function based on the *natural* gradient, constructed by rescaling the vanilla gradient based on the Fischer information matrix $\tilde{\nabla } = \mathbf {F}^{-1} \nabla _\theta J(\theta )$.

The expectation of the fitness function $f$ for a given sample $\mathbf {z}$ with respect to parameters $\theta$ is computed as

$$\begin{aligned} J(\theta ) = {\mathbb {E}}_\theta [f(\mathbf {z})] = \int f(\mathbf {z}) p(\mathbf {z}| \theta ) d\mathbf {z}\end{aligned}$$

where $p(\mathbf {z}| \theta )$ is a conditional probability distribution function given parameter $\theta$. This allows writing the updates for the distribution as

$$\begin{aligned} \theta \leftarrow \theta - \eta \tilde{\nabla }_\theta J = \theta - \eta \mathbf {F}^{-1} \nabla _\theta J(\theta ) \end{aligned}$$

The most representative algorithm of the family is Exponential NES (XNES; [14]), which maintains a multivariate Gaussian distribution over the parameters space, defined by the parameters $\theta = (\mu , \varSigma )$. Based on the equation above, with the addition of Monte Carlo estimation, fitness shaping and exponential local coordinates (see [46] for the full derivation), these parameters are updated as:

$$\begin{aligned}&\mu \leftarrow \mu + \eta _\mu \sum ^\lambda _{k=1} u_k \mathbf {z}_k \\&A \leftarrow A \exp \left( \frac{\eta _A}{2} \sum ^\lambda _{k=1} u_k (\mathbf {z}_k \mathbf {z}_k^\intercal - {\mathscr {I}})\right) \end{aligned}$$

with $\eta _\mu$ and $\eta _A$ learning rates, $\lambda$ number of estimation samples (the algorithm’s correspondent to population size), $u_k$ fitness shaping utilities, and *A* upper triangular matrix from the Choleski decomposition of $\varSigma$, $\varSigma = A^\intercal A$.

The update equation for $\varSigma$ bounds the performance to  with  number of parameters. At the time of its inception, this limited XNES to applications of few hundred dimensions. Leveraging modern hardware and libraries though, our current implementation easily runs on several thousands of parameters in minutes.^2^ Perhaps more importantly, its parametrization makes it a prime candidate for a GPU-based implementation, as long as $\theta$ can be maintained on GPU memory, with only the individuals being fetched at each generation.

#### Dynamically varying the dimensionality

This paper introduces a novel twist to the algorithm as the dimensionality of the distribution (and thus its parameters) varies during the run. Since the parameters are interpreted as network weights in *direct encoding* neuroevolution, changes in the network structure need to be reflected by the optimizer in order for future samples to include the new weights. Particularly, the multivariate Gaussian acquires new dimensions: $\theta$ should be updated keeping into account the order in which the coefficients of the distribution samples are inserted in the network topology.

In Sect. 3.3 we explain how the network update is carried through by initializing the new weights to zeros. In order to respect the network’s invariance, the expected value of the distribution ($\mu$) for the new dimension should be zero. As for $\varSigma$, we need values for the new rows and columns in correspondence to the new dimensions. We know that (i) the new weights did not vary so far in relation to the others (as they were equivalent to being fixed to zero until now), and that (ii) everything learned by the algorithm until now was based on the samples having always zeros in these positions. So $\varSigma$ must have for all new dimensions (i) zeros covariance and (ii) arbitrarily small variance (diagonal), only in order to bootstrap the search along these new dimensions.

Take for example a one-neuron feed-forward network with 2 inputs plus bias, totaling 3 weights. Let us select a function mapping the optimizer’s parameters to the weights in the network structure (i.e. the *genotype to phenotype* function), as to first fill the values of all input connections, then all bias connections. Extending the input size to 4 requires the optimizer to consider two more weights before filling in the bias:

$$\begin{aligned} \mu&= \begin{bmatrix} \mu _1&\mu _2&\mu _b \end{bmatrix} \quad \rightarrow \quad \begin{bmatrix} \mu _1&\mu _2&0&0&\mu _b \end{bmatrix}\\ \varSigma&= \begin{bmatrix} \sigma ^2_1 &{} \quad c _{12} &{} \quad c _{1b} \\ c _{21} &{} \quad \sigma ^2_2 &{} \quad c _{2b} \\ c _{b1} &{} \quad c _{b2} &{} \quad \sigma ^2_b \end{bmatrix} \quad \rightarrow \quad \begin{bmatrix} \sigma ^2_1 &{} \quad c _{12} &{} \quad 0 &{} \quad 0 &{} \quad c _{1b} \\ c _{21} &{} \quad \sigma ^2_2 &{} \quad 0 &{} \quad 0 &{} \quad c _{2b} \\ 0 &{} \quad 0 &{} \quad \epsilon &{} \quad 0 &{} \quad 0 \\ 0 &{} \quad 0 &{} \quad 0 &{} \quad \epsilon &{} \quad 0 \\ c _{b1} &{} \quad c _{b2} &{} \quad 0 &{} \quad 0 &{} \quad \sigma ^2_b \end{bmatrix} \end{aligned}$$

with $c _{ij}$ being the covariance between parameters *i* and *j*, $\sigma ^2_k$ the variance on parameter *k*, and $\epsilon$ being arbitrarily small (0.0001 here). The complexity of this step of course increases considerably with more sophisticated mappings, for example when accounting for recurrent connections and multiple neurons, but the basic idea stays the same. The evolution can pick up from this point on as if simply resuming, and learn how the new parameters influence the fitness.

## Experimental setup

The experimental setup further highlights the performance gain achieved, and is thus crucial to properly understand the results presented in the next section:

- All experiments were run on a single machine, using a 32-core Intel(R) Xeon(R) E5-2620 at 2.10GHz, with only 3GB of ram per core (including the Atari simulator and Python wrapper).
- The maximum run length on all games is capped to 200 interactions, meaning each agent is allotted a mere $1000$ frames, given our constant frameskip of 5.
- Population size and learning rates are dynamically adjusted based on the number of parameters, based on the XNES minimal population size and default learning rate [14]. We scale the population size by 1.5 and the learning rate by 0.5. In all runs on all games, the population size is between 18 and 42, again very limited in order to optimize run time on the available hardware.
- The dictionary growth is roughly controlled by $\delta$ (see Algorithm 1), but depends on the graphics of each game. The average dictionary size by the end of the run is around 30-50 centroids, but games with many small moving parts tend to grow over 100. In such games there seems to be direct correlation between higher dictionary size and performance, but our reference machine performed poorly over 150 centroids. We found numbers close to $\delta = 0.005$ to be robust in our setup across all games.
- Graphics resolution is reduced from $[210 \times 180 \times 3]$ to $[70 \times 80]$, averaging the color channels to obtain a gray-scale image. This also contributes to lower run times.
- Every individual is evaluated 5 times on 5 different simulator seeds to reduce fitness variance. Notably the most common method in the literature only requires a single game seed but a varying number of time-steps at game start before giving control to the agent (frameskip). We found this simple shifting however insufficient to overcome the sequence-learning ability of our RNN policies, especially since Neuroevolution training easily converges to local minima behaviors. We thereby foster improved generalization by choosing thereby 5 random seeds for the environment (same 5 throughout the learning), which correspond to 5 entirely independent “game trajectories”.
- Experiments are allotted a mere 100 generations, which averages to 2 to 3 hours of run time on our reference machine.

Those limitations were put in place to adapt to the available computational resources and to limit the run time. As a side-note, we ran experiments that show a minimal increase in score with less constraints settings, e.g., with Qbert we were able to reach a score of 1450 with unlimited time-steps, 300 generations and doubled population size. We ran experiments up to 1000 generations. The number of centroids however steadily increased in that case, until the quadratic complexity kicks in and slowed our progress to a crawl.

These computational restrictions are **extremely tight** compared to what is typically used in studies utilizing the ALE framework. Limited experimentation indicates that accessing the kind of hardware usually dedicated to modern deep learning consistently improves the results on the presented games. The code was written from scratch in Ruby and is fully available on GitHub under MIT license.^3^ ^4^

## Results

The goal of this work is not to propose a new generic feature extractor for Atari games, nor a novel approach to beat the best scores from the literature. The research for score optimization has recently surpassed even human scores, thanks to the 2020 publication of *Agent57* [2], the culmination of many years of work specifically on the Atari problem from multiple large teams, building on top of the advancements of a considerable number of previous papers. As a result the scores are extraordinary, but the cost in terms of complexity, optimization, model size and computational power for training are also disproportionate. Still, we include those results in Table 3 for reference, but it is crucial to understand how and why these numbers are not comparable.

Our work instead contributes to the field in the exactly opposite direction. Our declared goal is not to show how to improve Atari scores, but rather to demonstrate that an explicit separation of feature extraction and decision making enables addressing hard problems with much smaller resources and simplistic methods, while maintaining promising performance comparable to the top results in direct policy search, where the neural network is used to approximate the policy (i.e. neuroevolution) rather than the value function (i.e. Agent57). Table 2 emphasizes our findings in this regard.

Under these assumptions, Table 1 presents game scores over a set of 15 Atari games available on the ALE simulator. Here are the steps that led to such a selection: (i) games available through the OpenAI Gym; (ii) games with the same observation resolution of [210, 160] (uniquely for consistency); (iii) games not involving 3D perspective (as our simplistic feature extractor would be ineffective in that case). The resulting list was further narrowed down due to hardware limitations and available run time. A broader selection of games would support a broader applicability of our particular, specialized setup; our goal however is not to suggest its adoption, but to create a solid baseline upon which to build more effective feature extractors, highlighting that our simple setup is indeed able to play Atari games with competitive results.

To offer a more direct comparison, we opted for using the same settings as described above for all games, rather than delving into hyperparameter optimization. Some games performed well with these parameters (e.g. Phoenix); others feature many small moving parts in the observations, which would require a larger number of centroids for a proper encoding (e.g. Name This Game, Kangaroo); still others have complex dynamics, difficult to learn with such tiny networks (e.g. Demon Attack, Seaquest). Our setup is particularly suited to highlight these differences.

The resulting scores are compared with recent papers that offer a broad set of results across Atari games on comparable settings [8, 19, 38, 42]. Our list of games and correspondent results are available in Table 1. Scores were directly taken from the respective papers. Notably, our setup achieves high scores on *Qbert*, arguably one of the harder games for its requirement of strategic planning, confirming that the evolved policies are capable of some degree of sophistication.

The real results of the paper however are highlighted in Table 2, which compares the number of neurons, hidden layers and total connections utilized by each approach. Our setup uses up to two orders of magnitude fewer neurons, two orders of magnitude fewer connections, and is the only one using no hidden layer (only one layer of neurons, the output layer).

**Table 1:** Game scores. Scores (higher is better) on a sample of Atari games (sorted alphabetically) comparing our proposed approach of IDVQ $+$ DRSC $+$ XNES with results from HyperNeat [19] and OpenAI ES [38]. Results from GA (1B) [42] and NSRA-ES [8] are also provided to include work aimed at *expanding* the network size, rather than shrinking it (though the intersection between sets of games is limited). All methods were trained from scratch on raw pixel input (NSRA-ES uses a compact state representation read from the simulated Atari RAM to compute novelty). Column ‘*# neurs*’ indicates how many neurons were used in our work in a single layer (output) for each game. The number of neurons corresponds to the number of available actions in each game, i.e. no neurons are added for performance purpose.

Game	HyperNeat	OpenAI ES	GA (1B)	NSRA-ES	**Ours**	# neurs
Berzerk	1394	686	–	–	900	18
Bowling	135.8	30	–	–	82	6
DemonAttack	3590	1166.5	–	-	325	6
Enduro	93.6	95	60	–	7.4	9
FishingDerby	− 49	− 49	–	–	-10	18
Frostbite	2260	370	4536	3785	300	18
Gravitar	370	805	476	1140	1100	18
Kangaroo	800	11200	3790	–	1200	18
NameThisGame	6742	4503	–	–	920	6
Phoenix	1762	4041	–	–	4600	8
Qbert	695	147.5	–	1350	1250	6
Seaquest	716	1390	798	960	320	18
SpaceInvaders	1251	678.5	–	–	830	6
StarGunner	2720	1470	–	–	1200	18
TimePilot	7340	4970	–	–	4600	10

**Table 2:** Results. While our proposed approach of IDVQ + DRSC + XNES achieves not much lower scores (sometimes better; see Table 1), it does so using up to *two orders of magnitude* fewer neurons, and no hidden layers. The proposed feature extraction algorithm IDVQ+DRSC is simple enough (using basic, linear operations) to be arguably unable to contribute to the decision making process in a sensible manner (see Section 3.2.4). This implies that the tiny network trained on decision making alone is of sufficient complexity to learn a successful policy, potentially prompting for reconsidering the actual complexity of this standard benchmark. The number of neurons used in our approach solely depends on the size of each game actions space (see Table 1 for reference). The number of weights in our approach are scaled to the worst case of 150 centroids (1k for 6 neurons, 3k for 18 neurons), which in our runs was only reached by a few hard games: averages were more commonly in the 300 to 1k weights range

	HyperNeat	OpenAI ES	GA (1B)	NSRA-ES	Ours
# neurons	$\sim$ 3034	$\sim$ 650	$\sim$ 650	$\sim$ 650	6 – 18
# hidden layers	2	3	3	3	0
# connections	$\sim$ 906k	$\sim$ 436k	$\sim$ 436k	$\sim$ 436k	1k – 3k

## Discussion

It is important to note that the particular algorithm stack we have described in this paper does not work well on every possible visual problem, or even every Atari game. The IDVQ+DRSC pair was not designed as a general-purpose observation encoder, but as the simplest method that would work with the simplest games to remove the challenge of feature extraction, exposing the game’s control complexity to direct policy search. As such, it simply fails progressively as the game’s graphics become more convoluted. One can easily find counter-examples, such as games where large parts of the screen change with little correlation to the game state. This happens in games with forced screen scrolling, flashes and blinks, or simply with dynamic decorations playing on screen and unrelated to the game state. This limits the applicability of our proof-of-concept system to the games with the simplest graphics. It is However a limitation only in terms of implementation, which could easily be overcome by using a more generic (and sophisticated) encoder, now that the fundamental point put forward by this work has been made. The question, then, is whether a suitable form of pre-processing could be automatically learned or whether it should be selected for each game.

An obvious alternative for automatically learning pre-processing for new types of visual inputs is the autoencoder. Different types of autoencoders have been attempted for successfully learning state representation compressors for both 2D and 3D games [1, 17]. However, it is not clear that the utility of the autoencoder is much more general than the vector quantization-based approach we have used in this paper. For example, Alvernaz and Togelius co-trained autoencoders and policy networks to play *Doom*, and observed that the reconstruction error was higher when the walls in the game had more complex texture (such as bricks). This is because the autoencoder does not, on its own, know which aspects of the visual input are most relevant to preserve in the compressed representation. Creating a state compressor which takes the effect of the compressed representation on the policy network into account, perhaps by guiding compression with an error signal, is a major unsolved problem.

It should be noted that in addition to autoencoders and IDVQ, there are many other potential methods that could be used to compress observations for the purpose of reinforcement learning. In general, encoders can be rewarded for many other things than “simply” reconstructing the input, and several such methods have been proposed to create compressed visual state representations. This includes the evolutionary method described above [27], representations learned with inverse-dynamics objectives [33], representations rewarded for grouping functionally similar input images together [40], and representations that predict rewards [22]. Another simple but potentially useful way of preprocessing rich visual data is to simply downscale it [12]. It is not clear that any of these alternatives are to as well-suited to 2D games as IDVQ is. Nevertheless, this is a rich space which is worth exploring.

**Table 3:** **Comparison with the state of the art on Atari score optimization**. For reference, we include here the scores for the state of the art in Atari game playing optimizing for in-game scores: Agent57 [2], which was in 2020 the first implementation to surpass the reference “average human” performance on all 57 Atari games. The scores for “Average Human”, “Random“ and “Agent57” in the table are taken directly from the paper. Such an impressive achievement utilizes a classic reinforcement learning framework setup, where not one but two neural networks are used to approximate the *value function*, rather than the policy. As such, network size comparison is not directly applicable, though Figure 1 in their paper gives us an estimate (for both networks) of over 4’300 neurons and more than 1’600’000 weights. Notice that our method notably surpasses the random policy baseline on all games, supporting our “sensible play” thesis, and actually also overtakes reference human performance on one game: FishingDerby, using only 18 neurons

Game	Average human	Random	Agent57	**Ours**
Berzerk	2630	124	61508	900
Bowling	161	23	251	82
DemonAttack	1971	152	143161	325
Enduro	861	0	2368	7.4
FishingDerby	$-$39	$-$92	87	− 10
Frostbite	4335	65	541281	300
Gravitar	3351	173	19214	1100
Kangaroo	3035	52	24034	1200
NameThisGame	8049	2292	54387	920
Phoenix	7243	761	908264	4600
Qbert	13455	164	580329	1250
Seaquest	42055	68	999998	320
SpaceInvaders	1669	148	48681	830
StarGunner	10250	664	839574	1200
TimePilot	5229	3568	405425	4600

Another open question indirectly addressed in this work is to what extent limiting the size of the policy network offers any kind of remedy for the problem of overfitting in deep reinforcement learning. It has repeatedly been observed that deep reinforcement learning tends to learn very brittle solutions, in particular on the kind of third-person, two-dimensional representations that most Atari games offer. For example, we previously found that networks trained to particular games not only failed to generalize to other similar games, but also to different levels of the same game [24]. This could be mitigated to an extent by training on a large number of procedurally generated levels, but it did not seem possible to train a single network that could play arbitrary levels in a single game. In general, networks performed very badly on levels they were not trained, even if they were similar to levels in the training set. Similar results were found independently by [7]. One hypothesis is that the trained neural networks in this case simply act as lookup tables, matching particular scenes with stored actions. Even if that extreme hypothesis is not correct, it is clear that the trained networks overfitted considerably.

In supervised learning, it is standard practice to reduce the model size so as to avoid overfitting. The reason for this is that a function plus noise is generally more complex to describe than just the function, thus requiring a larger model. Smaller models by necessity can only implement simpler, potentially more fundamental functions. Therefore under the same training, whereas a huge deep network might overfit on a problem, a smaller network is much less likely to do so. The question is to what extent this applies to reinforcement learning as well. It stands to reason that a smaller network can only represent a simpler policy, and is therefore less able to overfit.

While the networks we produce with the method introduced in this paper are orders of magnitude smaller than deep networks, and could not reasonably implement very complex strategies, it is not clear that the system as a whole would be incapable of overfitting. In particular, the pre-processor creates visual features based on dynamic environmental stimuli. It could then be argued that it is the pre-processor that might suffer from overfitting, especially in the cases cited above where the stimuli is unrelated to the underlying game state. We have not investigated whether the current approach would overfit more or less than a more orthodox deep learning approach, or simply in a different way. However, we believe that the strategy of learning smaller policy networks holds promise for understanding overfitting in reinforcement learning in general.

Another potential advantage of small networks is interpretability. In principle it is very hard to interpret what has been learned when the policy is embedded in a neural network with thousands of neurons and hundreds of thousands of parameters; any understanding commonly comes from observing the behavior in new environments, or from techniques such as neural activation maximization. Networks with very few layers however—or just one, such as the networks presented here—are closely related to linear controllers, in our case deviating just because of the recurrent connections. This enables understanding the fundamental trend of the policy implementation more or less directly.

Our simplistic, explicit algorithms for feature extraction (IDVQ+DRSC) have the added advantage that it is easy to visualize the learned centroids, as proposed in Fig. 2. The centroids explicitly and directly represent the presence of determined sprites in fixed locations in the observation. The code generated from an observation is composed (by design: SC) of mostly zeros and few ones. The nonzero elements directly refers to the centroid-features identified as most prominent in the observation. The code is then directly fed as input to the policy network: each neuron will thus see a combination of weighted inputs, which are nonzero only in correspondence to the representative centroids (nonzero elements in the code).

By considering the relative weight magnitude from a nonzero code-entry to each of the action-neurons, it is possible to deduce the general policy trend of “when this sprite is present in the observation, prefer an action over the others”. For example, if the neuron are sorted as {up, left, right, down, jump, noop}, with a code having a single nonzero entry, and neural network weights from that one nonzero input to each neuron respectively are {0, 0.3, 0.4, 1.3, 1.0, 0.5}, then we can assume a bias of the network for selecting the up action when the sprite captured by the centroid of the nonzero input is representative of the observation.

Since there are also recurrent connection to take into account however, a more organic visualization of the full network would require highlighting dynamic changes in real time, and understand the impact of memory over time in biasing action selection. This would allow us to understand which parts of the network reacts to which input over time, and consequently produces which behavior.

## Conclusions

We presented a method to address complex learning tasks such as learning to play Atari games by decoupling policy learning from feature construction, learning each independently but simultaneously to further specialization. Features are extracted from raw pixel observations coming from the game using a novel and efficient sparse coding algorithm named Direct Residual Sparse Coding. The resulting compact code is based on a dictionary trained online with yet another new algorithm called Increasing Dictionary Vector Quantization, which uses the observations obtained on-line by the networks’ interacting with the environment as the policy search progresses. Finally, tiny neural networks are evolved to decide actions based on the encoded observations, to achieve results comparable with the deep neural networks typically used for these problems while being *two orders of magnitude* smaller.

Our work shows how a relatively simple and efficient feature extraction method, which counter-intuitively does not use reconstruction error for training, can effectively extract meaningful features from a range of different games. On top of that, the neural network trained for policy approximation is also very small in size, showing that the decision making itself can be done by relatively simple functions. The implication is that both feature extraction and actual control on some Atari games are not as complex as often considered, with much of the difficulty coming from addressing the two sides of the problem at the same time using end-to-end training.

We empirically evaluated our method on a set of well-known Atari games using the ALE benchmark in the OpenAI Gym framework. Tight performance restrictions are posed on these evaluations, which can run on common personal computing hardware as opposed to the large server farms often used for deep reinforcement learning research. The source code is open-sourced for further reproducibility. The game scores are comparable with the state of the art in neuroevolution, while using but a minimal fraction of the computational resources usually devoted to this task. One goal of this paper is to clear the way for new approaches to learning, and to call into question a certain orthodoxy in deep reinforcement learning, namely that image processing and policy should be learned together *(end-to-end)*.

As future work, we plan to identify the actual complexity required to achieve top scores on a (broader) set of games. This requires first applying a feature extraction method with state-of-the-art performance, such as based on *autoencoders*. Our findings though support the design of novel variations focused on state differentiation rather than reconstruction error minimization. As for the decision maker, the natural next step is to train deep networks entirely dedicated to policy learning, capable in principle of scaling to problems of unprecedented complexity. Training large, complex networks with neuroevolution requires further investigation in scaling sophisticated evolutionary algorithms to higher dimensions. An alternative research direction considers the application of deep reinforcement learning methods on top of the external feature extractor. Finally, a straightforward direction to improve scores is simply to release the constraints on available performance: longer runs, optimized code and parallelization should still find room for improvement even using our current, minimal setup.

## Footnotes

1 This has also been empirically verified in earlier iterations of this work

2 For a NES algorithm suitable for evolving deep neural networks see Block Diagonal NES [9], which scales linearly on the number of neurons / layers.

3 Experiments and environment wrappers: https://github.com/giuse/DNE.

4 Methods and tools: https://github.com/giuse/machine_learning_workbench.

## References

[1] Alvernaz, S., & Togelius, J. (2017). Autoencoder-augmented neuroevolution for visual doom playing. In *Computational Intelligence and Games (CIG)*, 2017 IEEE Conference on, IEEE, pp 1–8.

[2] Badia, A. P., Piot, B., Kapturowski, S., Sprechmann, P., Vitvitskyi, A., Guo, D., & Blundell, C. (2020). Agent57: Outperforming the Atari human benchmark. arXiv preprint arXiv:200313350.

[3] Bellemare MG, Naddaf Y, Veness J, Bowling M (2013). The arcade learning environment: An evaluation platform for general agents. Journal of Artificial Intelligence Research.

[4] Brockman, G., Cheung, V., Pettersson, L., Schneider, J., Schulman, J., Tang, J., & Zaremba, W. (2016). Openai gym. arXiv:1606.01540.

[5] Chrabaszcz, P., Loshchilov, I., & Hutter, F. (2018). Back to basics: Benchmarking canonical evolution strategies for playing atari. arXiv preprint arXiv:180208842.

[6] Coates, A., & Ng, A. Y. (2011). The importance of encoding versus training with sparse coding and vector quantization. In *Proceedings of the 28th International Conference on Machine Learning (ICML-11)*, pp 921–928.

[7] Cobbe, K., Klimov, O., Hesse, C., Kim, T., & Schulman, J. (2018). Quantifying generalization in reinforcement learning. arXiv preprint arXiv:181202341.

[8] Conti, E., Madhavan, V., Such, F. P., Lehman, J., Stanley, K., & Clune, J. (2018). Improving exploration in evolution strategies for deep reinforcement learning via a population of novelty-seeking agents. *Advances in Neural Information Processing Systems (NIPS),* 5032–5043.

[9] Cuccu, G., & Gomez, F. (2012). Block diagonal natural evolution strategies. In *International Conference on Parallel Problem Solving from Nature*, Springer, pp 488–497.

[10] Cuccu, G., Luciw, M., Schmidhuber, J., & Gomez, F. (2011). Intrinsically motivated neuroevolution for vision-based reinforcement learning. In *Development and Learning (ICDL), 2011 IEEE International Conference on, IEEE*, vol 2, pp 1–7.

[11] Cuccu, G., Togelius, J., & Cudré-Mauroux, P. (2019). Playing Atari with six neurons. In *Proceedings of the 18th International Conference on Autonomous Agents and MultiAgent Systems, International Foundation for Autonomous Agents and Multiagent Systems*, pp 998–1006.

[12] Ecoffet, A., Huizinga, J., Lehman, J., Stanley, K. O., & Clune, J. (2019). Go-explore: a new approach for hard-exploration problems. arXiv preprint arXiv:190110995.

[13] Floreano D, Dürr P, Mattiussi C (2008). Neuroevolution: from architectures to learning. Evolutionary Intelligence.

[14] Glasmachers, T., Schaul, T., Yi, S., Wierstra, D., & Schmidhuber, J. (2010). Exponential natural evolution strategies. In *Proceedings of the 12th annual conference on Genetic and evolutionary computation*, ACM, pp 393–400.

[15] Gomez F, Schmidhuber J, Miikkulainen R (2008). Accelerated neural evolution through cooperatively coevolved synapses. Journal of Machine Learning Research.

[16] Gray R (1984). Vector quantization. IEEE ASSP Magazine.

[17] Ha, D., & Schmidhuber, J. (2018). World models. arXiv preprint arXiv:180310122.

[18] Hansen N, Ostermeier A (2001). Completely derandomized self-adaptation in evolution strategies. Evolutionary Computation.

[19] Hausknecht M, Lehman J, Miikkulainen R, Stone P (2014). A neuroevolution approach to general Atari game playing. IEEE Transactions on Computational Intelligence and AI in Games.

[20] Hessel, M., Modayil, J., Van Hasselt, H., Schaul, T., Ostrovski, G., Dabney, W., Horgan, D., Piot, B., Azar, M., & Silver, D. (2017). Rainbow: Combining improvements in deep reinforcement learning. arXiv preprint arXiv:171002298.

[21] Igel, C. (2003). Neuroevolution for reinforcement learning using evolution strategies. In *Evolutionary Computation, 2003. CEC’03. The 2003 Congress on, IEEE*, vol 4, pp 2588–2595.

[22] Jaderberg, M., Mnih, V., Czarnecki, W. M., Schaul, T., Leibo, J. Z., Silver, D., & Kavukcuoglu, K. (2016). Reinforcement learning with unsupervised auxiliary tasks. arXiv preprint arXiv:161105397.

[23] Juliani, A., Khalifa, A., Berges, V. P., Harper, J., Henry, H., Crespi, A., Togelius, J., & Lange, D. (2019). Obstacle tower: A generalization challenge in vision, control, and planning. arXiv preprint arXiv:190201378.

[24] Justesen, N., Torrado, R. R., Bontrager, P., Khalifa, A., Togelius, J., & Risi, S. (2018). Illuminating generalization in deep reinforcement learning through procedural level generation. In NeurIPS Workshop on Deep Reinforcement Learning.

[25] Justesen, N., Bontrager, P., Togelius, J., & Risi, S. (2019). Deep learning for video game playing. IEEE Transactions on Games.

[26] Kempka, M., Wydmuch, M., Runc, G., Toczek, J., & Jaśkowski, W. (2016). Vizdoom: A doom-based ai research platform for visual reinforcement learning. In *2016 IEEE Conference on Computational Intelligence and Games (CIG)*, IEEE, pp 1–8.

[27] Koutník, J., Schmidhuber, J., & Gomez, F. (2014). Evolving deep unsupervised convolutional networks for vision-based reinforcement learning. In *Proceedings of the 2014 Annual Conference on Genetic and Evolutionary Computation*, pp 541–548.

[28] Li, C., Farkhoor, H., Liu, R., & Yosinski, J. (2018). Measuring the intrinsic dimension of objective landscapes. arXiv preprint arXiv:180408838.

[29] Mairal, J., Bach, F., Ponce, J., et al. (2014). Sparse modeling for image and vision processing. *Foundations and Trends*$$\textregistered$$*in Computer Graphics and Vision,**8*(2–3), 85–283.

[30] Mallat SG, Zhang Z (1993). Matching pursuits with time-frequency dictionaries. IEEE Transactions on Signal Processing.

[31] Mnih V, Kavukcuoglu K, Silver D, Rusu AA, Veness J, Bellemare MG, Graves A, Riedmiller M, Fidjeland AK, Ostrovski G (2015). Human-level control through deep reinforcement learning. Nature.

[32] Ontanón S, Synnaeve G, Uriarte A, Richoux F, Churchill D, Preuss M (2013). A survey of real-time strategy game AI research and competition in starcraft. IEEE Transactions on Computational Intelligence and AI in Games.

[33] Pathak, D., Agrawal, P., Efros, A. A., & Darrell, T. (2017). Curiosity-driven exploration by self-supervised prediction. In *Proceedings of the IEEE Conference on Computer Vision and Pattern Recognition Workshops*, pp 16–17.

[34] Pati, Y. C., Rezaiifar, R., & Krishnaprasad, P. S. (1993). Orthogonal matching pursuit: Recursive function approximation with applications to wavelet decomposition. In *Signals, Systems and Computers, 1993. 1993 Conference Record of The Twenty-Seventh Asilomar Conference on, IEEE*, pp 40–44.

[35] Perez, D., Liu, J., Abdel, Samea Khalifa A., Gaina, R. D., Togelius, J., & Lucas, S. M. (2019). General video game AI: a multi-track framework for evaluating agents, games and content generation algorithms. IEEE Transactions on Games.

[36] Perez-Liebana, D., Samothrakis, S., Togelius, J., Schaul, T., & Lucas, S.M. (2016). General video game AI: Competition, challenges and opportunities. In *Thirtieth AAAI Conference on Artificial Intelligence*.

[37] Risi S, Togelius J (2017). Neuroevolution in games: State of the art and open challenges. IEEE Transactions on Computational Intelligence and AI in Games.

[38] Salimans, T., Ho, J., Chen, X., Sidor, S., & Sutskever, I. (2017). Evolution strategies as a scalable alternative to reinforcement learning. arXiv preprint arXiv:170303864.

[39] Schaul, T., Glasmachers, T., & Schmidhuber, J. (2011). High dimensions and heavy tails for natural evolution strategies. In *Proceedings of the 13th Annual Conference on Genetic and Evolutionary Computation, ACM*, pp 845–852.

[40] Sermanet, P., Lynch, C., Chebotar, Y., Hsu, J., Jang, E., Schaal, S., & Levine, S. (2018). Time-contrastive networks: Self-supervised learning from video. In *2018 IEEE International Conference on Robotics and Automation (ICRA), IEEE*, pp 1134–1141.

[41] Stanley KO, Miikkulainen R (2002). Evolving neural networks through augmenting topologies. Evolutionary Computation.

[42] Such, F. P., Madhavan, V., Conti, E., Lehman, J., Stanley, K. O., & Clune, J. (2017). Deep neuroevolution: Genetic algorithms are a competitive alternative for training deep neural networks for reinforcement learning. arXiv preprint arXiv:171206567

[43] Togelius J, Schaul T, Wierstra D, Igel C, Gomez F, Schmidhuber J (2009). Ontogenetic and phylogenetic reinforcement learning. Künstliche Intelligenz.

[44] Togelius J, Shaker N, Karakovskiy S, Yannakakis GN (2013). The mario AI championship 2009–2012. AI Magazine.

[45] Vinyals, O., Ewalds, T., Bartunov, S., Georgiev, P., Vezhnevets, A. S., Yeo, M., Makhzani, A., Küttler, H., Agapiou, J., Schrittwieser, J., et al. (2017). Starcraft II: a new challenge for reinforcement learning. arXiv preprint arXiv:170804782.

[46] Wierstra, D., Schaul, T., Peters, J., & Schmidhuber, J. (2008). Natural evolution strategies. In *Evolutionary Computation, 2008. CEC 2008.(IEEE World Congress on Computational Intelligence). IEEE Congress on, IEEE*, pp 3381–3387.

[47] Wierstra D, Schaul T, Glasmachers T, Sun Y, Peters J, Schmidhuber J (2014). Natural evolution strategies. Journal of Machine Learning Research.

[48] Yannakakis, G. N., & Togelius, J. (2018). Artificial Intelligence and Games. Springer, http://gameaibook.org.

[49] Yao X (1999). Evolving artificial neural networks. Proceedings of the IEEE.

[50] Zhang Z, Xu Y, Yang J, Li X, Zhang D (2015). A survey of sparse representation: algorithms and applications. IEEE Access.

